# CapsNet-SSP: multilane capsule network for predicting human saliva-secretory proteins

**DOI:** 10.1186/s12859-020-03579-2

**Published:** 2020-06-09

**Authors:** Wei Du, Yu Sun, Gaoyang Li, Huansheng Cao, Ran Pang, Ying Li

**Affiliations:** 1 Key Laboratory of Symbol Computation and Knowledge Engineering of the Ministry of Education, College of Computer Science and Technology, Jilin University, Changchun, 130012 China; 2 Center for Fundamental and Applied Microbiomics, Biodesign Institute, Arizona State University, Tempe, AZ 85287 USA

**Keywords:** Saliva-secretory protein, Deep learning, Capsule network, Convolutional neural network

## Abstract

**Background:**

Compared with disease biomarkers in blood and urine, biomarkers in saliva have distinct advantages in clinical tests, as they can be conveniently examined through noninvasive sample collection. Therefore, identifying human saliva-secretory proteins and further detecting protein biomarkers in saliva have significant value in clinical medicine. There are only a few methods for predicting saliva-secretory proteins based on conventional machine learning algorithms, and all are highly dependent on annotated protein features. Unlike conventional machine learning algorithms, deep learning algorithms can automatically learn feature representations from input data and thus hold promise for predicting saliva-secretory proteins.

**Results:**

We present a novel end-to-end deep learning model based on multilane capsule network (CapsNet) with differently sized convolution kernels to identify saliva-secretory proteins only from sequence information. The proposed model CapsNet-SSP outperforms existing methods based on conventional machine learning algorithms. Furthermore, the model performs better than other state-of-the-art deep learning architectures mostly used to analyze biological sequences. In addition, we further validate the effectiveness of CapsNet-SSP by comparison with human saliva-secretory proteins from existing studies and known salivary protein biomarkers of cancer.

**Conclusions:**

The main contributions of this study are as follows: (1) an end-to-end model based on CapsNet is proposed to identify saliva-secretory proteins from the sequence information; (2) the proposed model achieves better performance and outperforms existing models; and (3) the saliva-secretory proteins predicted by our model are statistically significant compared with existing cancer biomarkers in saliva. In addition, a web server of CapsNet-SSP is developed for saliva-secretory protein identification, and it can be accessed at the following URL: http://www.csbg-jlu.info/CapsNet-SSP/. We believe that our model and web server will be useful for biomedical researchers who are interested in finding salivary protein biomarkers, especially when they have identified candidate proteins for analyzing diseased tissues near or distal to salivary glands using transcriptome or proteomics.

## Background

Saliva comes mainly from three pairs of salivary glands (parotid, submandibular and sublingual) and numerous minor salivary glands spread throughout the oral cavity [1]. Similar to other human body fluids, saliva is rich in biomolecules that are secreted by the salivary glands or that leak from nearby tissues [2]. Furthermore, biomolecules are released into the blood circulation system by various organs throughout the body and then secreted into saliva [3]. Thus, the biomolecules in saliva can reflect the health of specific organs, including organs near the salivary glands and distal organs. Previous studies have shown that certain biomolecules may appear in saliva when the patients have oral cancer [4, 5], head and neck squamous-cell carcinoma (HNSCC) [6], breast cancer [7], prostate cancer [8] and lung cancer [9].

Accurately measuring and evaluating biomarkers as indicators for differentiating normal and disease samples [10] is important for detecting diseases, making prognoses, and studying disease occurrence mechanisms [11]. At present, biomarker detection from body fluids such as blood, urine and saliva is an effective method for diagnosing diseases [12–16]. Zhang et al. recently proposed a series of prediction methods for secretory proteins and them in plasma, which play a great role in the diagnosis of early cancer and other diseases [17, 18]. With the rapid development of *omics* technology, researchers have acquired vast amounts of disease-related data. Therefore, it is desirable to use bioinformatics methods to identify highly sensitive and specific biomarkers from big data. Because there are many signals for various physiological and pathophysiological conditions in blood, most studies on body fluid biomarkers focus on blood biomarkers [19, 20]. However, saliva is a better source of biomarkers because it is relatively simple in composition and can be easily and noninvasively collected [21].

Many studies have identified biomarkers of various diseases in saliva based on proteomics experiments by performing comparative proteomic analyses of saliva samples from patients with specific diseases and control groups [3, 4, 22]. However, comparing and quantifying proteome data from saliva samples is challenging due to the large number of sparsely occurring proteins in the saliva, have large dynamic ranges [3]. Due to the limitations of proteomic experimental techniques, the discovery of saliva biomarkers faces many difficulties [23]. Developing a method of accurately predicting human saliva-secretory proteins could solve these problems to some extent. However, few studies have focused on establishing a computational model for predicting human saliva-secretory proteins. The existing studies are based on conventional machine learning methods in which features are selected from feature sets and then classifiers are constructed from training sets [16, 24]. Therefore, the results of these methods are largely dependent on the selected features. Compared with conventional machine learning techniques, deep learning methods can automatically learn complex feature representations from raw data [25].

**Fig. 1:**
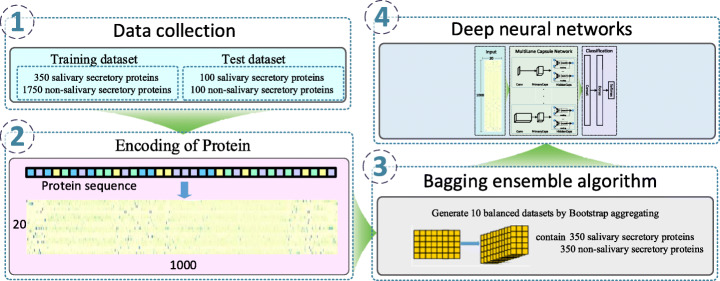
The flowchart for predicting human saliva-secretory proteins

In this study, we propose an end-to-end prediction model based on a deep learning framework that consists mainly of a multilane Capsule Network with differently sized convolution kernels. Our model can accurately identify human saliva-secretory proteins based on only sequence information. A flowchart of our model is shown in Fig. 1. Saliva-secretory protein identification is formulated as a binary classification problem in which each protein is classified as either a saliva-secretory protein or not. The first step involves converting the input protein sequences into evolutionary profile matrices using the Position-specific Iterative Basic Local Alignment Search Tool (PSI-BLAST). To address imbalance issues in the dataset during the training process, the bagging ensemble learning method is applied to the training set. Then, the evolutionary profile matrices of the training set are input into the proposed model to train the model parameters. The proposed model achieves high accuracies using 10-fold cross-validation on the training set and an independent test set (0.905 on training set; 0.888 on independent test set), thus outperforming existing methods [24] based on traditional machine learning algorithms. By comparing human saliva-secretory proteins detected experimentally by other studies with the results of our model, we find that our model can achieve a true positive rate of 89%. By comparing known salivary protein biomarkers of cancer with the results of our model, we find that our model can achieve an average true positive rate of 88%. A web server is developed for predicting saliva-secretory proteins, which can be accessed at the following URL: http://www.csbg-jlu.info/CapsNet-SSP/. We believe that our predictive model and web server are useful for biomedical researchers interested in finding protein biomarkers in saliva, especially when they have candidate proteins for analyzing diseased tissues near or distal to salivary glands using transcriptome or proteome data. The main contributions of this paper are as follows: (1) a deep-learning-based end-to-end prediction model for identifying saliva-secretory proteins solely from sequence information is proposed; (2) the proposed model performs well and outperforms existing methods; and (3) saliva-secretory protein identification is statistically significant for existing cancer biomarkers in saliva.

## Results

### Performance measurements

To compare the performances of different prediction models, the accuracy, sensitivity, specificity, F-score, Matthews correlation coefficient (MCC), and AUC are applied as evaluation metrics. The corresponding formulas are as follows:

1 $$ \mathrm{accuracy}=\frac{TP+ TN}{N_{total}} $$

2 $$ \mathrm{sensitivity}=\mathrm{recall}=\frac{TP}{TP+ FN} $$

3 $$ \mathrm{specificity}=\frac{TN}{TN+ FP} $$

4 $$ \mathrm{precision}=\frac{TP}{TP+ FP} $$

5 $$ \mathrm{F}\hbox{-} \mathrm{score}=2\cdot \frac{\mathrm{precision}\cdot \mathrm{recall}}{\mathrm{precision}+\mathrm{recall}} $$

6 $$ \mathrm{MCC}=\frac{\left( TP\times TN- FP\times FN\right)}{\sqrt{\left( TP+ FN\right)\left( TP+ FP\right)\left( TN+ FP\right)\left( TN+ FN\right)}}, $$

where *TP* is the number of true positives, *TN* is the number of true negatives, *FP* is the number of false positives, *FN* is the number of false negatives, and *N*_*total*_ is the number of total samples in the validation or test set. In machine learning, the MCC is used as a measure of the quality of binary classifications [26] and has a value between − 1 and + 1, where + 1 represents perfect classification, 0 represents random classification and − 1 signifies total disagreement between prediction and observation. Since accuracy, sensitivity, specificity, precision, F-score and MCC are threshold-dependent, a threshold needs to be selected for calculating the specific value. In evaluating binary classifications, MCC produces more informative and truthful scores than accuracy and F-score [27]. Therefore, in this article, the threshold is set where the MCC reaches the maximum value. The ROC curve is a graphical plot that illustrates the classification ability of a binary classifier by plotting the true positive rate (TPR) against the false positive rate (FPR) with various discrimination thresholds [28]. When using normalized units, the AUC can be between 0 and 1 and represents the probability that a classifier will rank a randomly chosen positive instance higher than a randomly chosen negative instance. In brief, a larger AUC value indicates better performance. The precision-recall curve is a plot of precision and recall with various discrimination thresholds.

### Evaluating the performance of CapsNet-SSP

**Fig. 2:**
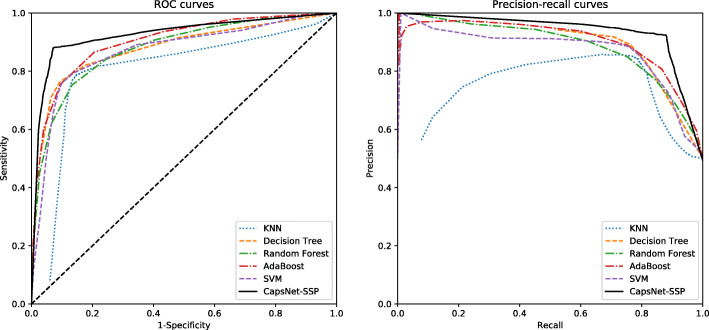
ROC curves and precision-recall curves of different methods on the training dataset

To evaluate the performance of CapsNet-SSP, we first generate balanced datasets using bagging ensemble learning. In each iteration of bagging ensemble learning, 350 saliva-secretory proteins and 350 non-saliva-secretory proteins are used to evaluate the performance of the model using 10-fold cross-validation. The performance distribution of the bagging iterations ranges from 0.859 to 0.906 for identifying saliva-secretory proteins and from 0.909 to 0.947 for identifying non-saliva-secretory proteins, which is generally desirable. The average accuracy, sensitivity, specificity, precision, F-score, MCC and AUC values for 10 iterations of the bagging ensemble learning approach are 0.905, 0.880, 0.929, 0.924, 0.902, 0.810 and 0.930, respectively. To evaluate the performance of CapsNet-SSP against methods based on conventional machine learning, we test all the models on the same dataset. The performance metrics of CapsNet-SSP and other methods are listed in Table 1; the SVM method was proposed by Sun et al [24]. To ensure a comprehensive and systematic comparison, we also construct several other prediction models, including k-nearest neighbor (KNN), decision tree, random forest and adaptive boosting (AdaBoost) based on the selected features in [24]. The average ROC and precision-recall curves for different methods are plotted in Fig. 2. According to Table 1 and Fig. 2, CapsNet-SSP yields the best predictive performance.

**Table 1:** The performances of CapsNet-SSP and other methods on the training set

Methods	Accuracy	Sensitivity	Specificity	Precision	F-score	MCC	AUC
KNN	0.835	0.763	0.907	0.891	0.822	0.677	0.878
Decision Tree	0.820	0.789	0.852	0.842	0.815	0.642	0.800
Random Forest	0.810	0.752	0.867	0.850	0.798	0.623	0.879
AdaBoost	0.830	0.754	0.905	0.889	0.836	0.667	0.905
SVM	0.830	0.760	0.899	0.883	0.822	0.666	0.877
**CapsNet-SSP**	**0.905**	**0.880**	**0.929**	**0.924**	**0.902**	**0.810**	**0.930**

The threshold is set where the MCC reaches the maximum value

**Fig. 3:**
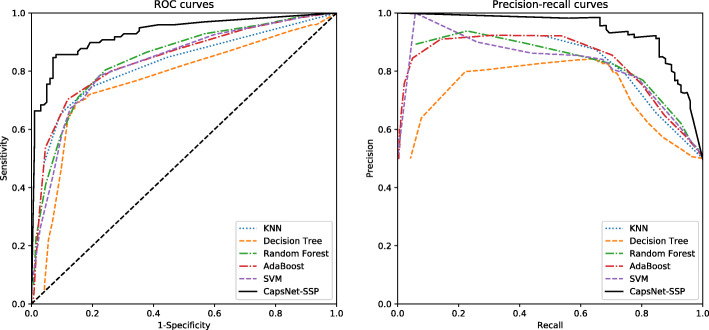
ROC curves and precision-recall curves of different methods on the independent test set

In addition, to evaluate the performance of the proposed model on the test set, we train the proposed model using bagging ensemble learning over 10 iterations. In each iteration, the models are trained with 350 saliva-secretory proteins and 350 non-saliva-secretory proteins, and the performance of the trained model is evaluated on an independent test set containing 100 saliva-secretory proteins and 100 non-saliva-secretory proteins. Then, the final prediction results are calculated by averaging the results from the 10 iterations. The performance metrics of CapsNet-SSP and other methods are shown in Table 2. The average accuracy, sensitivity, specificity, precision, F-score, MCC and AUC values of CapsNet-SSP on the independent test set are 0.888, 0.847, 0.929, 0.922, 0.884, 0.779 and 0.948, respectively. The ROC curves and precision-recall curves are illustrated in Fig. 3, showing that the performance of CapsNet-SSP is better than those of other methods. Regarding the independent test set, our model performs better than the methods based on conventional machine learning.

**Table 2:** The performances of CapsNet-SSP and other methods on the independent test set

Methods	Accuracy	Sensitivity	Specificity	Precision	F-score	MCC	AUC
KNN	0.778	0.649	0.907	0.875	0.745	0.575	0.809
Decision Tree	0.772	0.692	0.851	0.823	0.752	0.550	0.740
Random Forest	0.781	0.804	0.758	0.769	0.786	0.563	0.836
AdaBoost	0.792	0.703	0.881	0.855	0.772	0.593	0.847
SVM	0.781	0.784	0.778	0.779	0.782	0.562	0.857
**CapsNet-SSP**	**0.888**	**0.847**	**0.929**	**0.922**	**0.884**	**0.779**	**0.948**

The threshold is set where the MCC reaches the maximum value

### Comparing the performances of different deep learning architectures

**Fig. 4:**
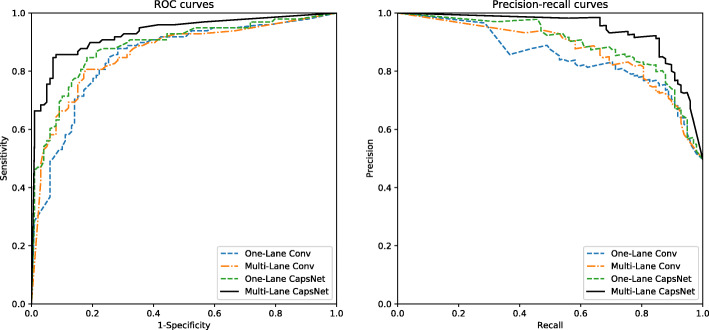
The ROC curves and precision-recall curves using different architectures in CapsNet-SSP

To better evaluate the contributions of the different architectures in the model, we compare the performances of different architectures on the independent test set by bagging ensemble learning. The results of performance comparison are shown in Table 3 and Fig. 4. First, the architecture using only the convolutional layer with 10 kernels of size 3 (One-Lane Conv) achieves average accuracy, sensitivity, specificity, precision, F-score, MCC and AUC scores of 0.797, 0.878, 0.717, 0.755, 0.811, 0.602 and 0.863, respectively. After adding the PrimaryCaps layer and the HiddenCaps layer (One-Lane CapsNet), the performance improves substantially. Moreover, comparing the performance results between the architecture of CapsNet with one lane (One-Lane CapsNet) and that with multilane CapsNet, the model with multilane CapsNet improve the performance greatly. To further evaluate whether the improvement of multilane CapsNet is significant or not, we calculate the *p*-values between multilane CapsNet and other architectures using the paired t-test [29] on the results of ten iterations of the bagging algorithm. The *p*-values are shown in brackets in Table 3. As shown in Table 3 and Fig. 4, the performance of the architecture of multilane CapsNet is statistically superior to the other architectures in predicting saliva-secretory proteins.

**Table 3:** Performance comparison using different architectures in CapsNet-SSP

Architectures	Accuracy	Sensitivity	Specificity	Precision	F-score	MCC	AUC
One-Lane Conv	0.792 (8.8e-08)	0.827 (5.0e-07)	0.758 (2.3e-07)	0.771 (4.0e-08)	0.811 (3.6e-07)	0.602 (1.6e-07)	0.863 (1.1e-06)
Multi-Lane Conv	0.812 (4.4e-08)	0.806 (6.4e-06)	0.818 (1.0e-07)	0.814 (1.0e-08)	0.810 (9.6e-07)	0.624 (4.1e-08)	0.869 (2.5e-06)
One-Lane CapsNet	0.832 (0.015)	0.847 (0.04)	0.818 (0.013)	0.822 (0.013)	0.834 (0.013)	0.665 (0.025)	0.915 (0.001)
**Multi-Lane CapsNet**	**0.888** (N/A)	**0.847** (N/A)	**0.929** (N/A)	**0.922** (N/A)	**0.884** (N/A)	**0.779** (N/A)	**0.948** (N/A)

The threshold is set where the MCC reaches the maximum value, and the values in brackets are *p*-values

**Fig. 5:**
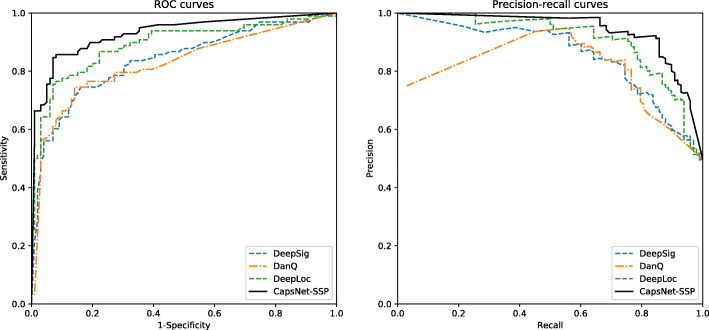
The ROC curves and precision-recall curves of different deep learning architectures

To better evaluate the performance of the proposed model, we compare our model with existing deep learning architectures on an independent test set. There is growing interest in applying deep learning methods to biological sequence analysis. For example, a convolutional neural network (CNN) is used in DeepSig to detect signal peptides in proteins [30]. DanQ uses a hybrid of CNN and a bidirectional long short-term memory network (BLSTM) to predict the properties and functions of DNA sequences [31]. An end-to-end model based on CNN, BLSTM and an attention mechanism is used in DeepLoc to predict protein subcellular localization [32]. To ensure a fair comparison with other deep learning architectures, we use the same balanced datasets and training strategy as our proposed model CapsNet-SSP to train these deep learning models. Specifically, we use the deep learning architectures proposed in DeepSig [30], DanQ [31] and DeepLoc [32] to replace our network architecture in the code, respectively. The performance comparison results of different deep learning architectures are shown in Table 4, and the average ROC and precision-recall curves for different models are plotted in Fig. 5. To further evaluate whether the improvement of CapsNet-SSP is significant or not, we calculate the *p*-values between CapsNet-SSP and other deep learning architectures using the paired t-test [29] on the results of ten iterations of the bagging algorithm. The *p*-values are shown in brackets in Table 4. As shown in Table 4 and Fig. 5, the performance of CapsNet-SSP is significantly superior to those of the other deep learning architectures on the independent test set.

**Table 4:** Performance comparison of deep learning architectures

Architectures	Accuracy	Sensitivity	Specificity	Precision	F-score	MCC	AUC
DeepSig	0.792 (0.011)	0.745 (0.030)	0.838 (0.009)	0.820 (0.016)	0.781 (7.8e-04)	0.586 (0.011)	0.867 (5.8e-07)
DanQ	0.802 (1.4e-05)	0.745 1.8e-05)	0.859 (3.5e-05)	0.839 (3.3e-05)	0.789 (2.2e-05)	0.608 (1.8e-05)	0.886 (6.3e-06)
DeepLoc	0.843 (0.013)	0.755 (0.029)	0.929 (0.037)	0.914 (0.038)	0.827 (0.016)	0.695 (0.013)	0.891 (0.015)
**CapsNet-SSP**	**0.888** **(N/A)**	**0.847** **(N/A)**	**0.929** **(N/A)**	**0.922** **(N/A)**	**0.884** **(N/A)**	**0.779** **(N/A)**	**0.948** **(N/A)**

The threshold is set where the MCC reaches the maximum value, and the values in brackets are *p*-values

### Comparing the performances of different strategies for class imbalance

Ensemble learning techniques, including bagging-based algorithm, boosting-based algorithmand and hybrid-based algorithm, can solve the problem of class imbalance [33]. To compare the performance of these strategies, first we train the proposed model without any strategy for solving the class imbalance problem. Then, we evaluate the performance of boosting-based and hybrid-based algorithms on the independent test set. The boosting-based and hybrid-based algorithms are implemented using RUSBoost [34] and EasyEnsemble [35], respectively. The performance metrics of different strategies on the independent test set are shown in Table 5. From the table, we can see that the performances of all the strategies based on ensemble learning techniques for the class imbalance problem are improved. Among them, the performance improvement of the bagging-based method is the most significant.

**Table 5:** Comparison of the performances of different strategies for class imbalance

Strategies	Accuracy	Sensitivity	Specificity	Precision	F-score	MCC	AUC
No strategy	0.853	0.796	0.909	0.897	0.843	0.710	0.916
Hybrid-based	0.868	0.857	0.879	0.875	0.866	0.736	0.939
Boosting-based	0.868	0.827	0.909	0.900	0.862	0.738	0.918
Bagging-based	0.888	0.847	0.929	0.922	0.884	0.779	0.948

The threshold is set where the MCC reaches the maximum value

### Predicting and ranking human saliva-secretory proteins

We rank 20,186 human proteins reviewed in the Universal Protein Resource (UniProt) database [36] using the *S*-value, which is defined as follows:

7 $$ S=2\times \left(\arg \max (p)-0.5\right)\times \max (p), $$

where *p* is the result of the softmax output from CapsNet-SSP and arg max(*p*) (0, 1) represents the indices of the maximum values. Then, to rank the human saliva-secretory proteins that do not overlap with our training set, 3449 proteins in saliva are collected using the LC-MS/MS analyses reported in the literature [37–42], of which 182 are detected in all studies. Of these 182 proteins, 87 are secretory proteins in SPD [43], LOCATE [44] and UniProt [36]. Next, we remove the proteins in the training set from the 87 proteins and obtain the 37 proteins shown in Additional file 1.

Table 6 shows the ranking results of these 37 proteins using the model based on SVM [24], DeepLoc [32] and CapsNet-SSP. Of the 37 proteins, 16 (43.24%), 24 (64.86%), 29 (75.68%) and 33 (89.19%) are ranked among the top 1000, 2000, 3000 and 4000 using CapsNet-SSP, respectively. We count the results of these saliva proteomics studies [37–42] and find that the maximum number of salivary proteins does not exceed 4000. Consequently, we use 4000 as the maximum number of saliva-secretory proteins. In the results corresponding to the models based on SVM and DeepLoc, 3, 7, 7, and 9 proteins and 5, 19, 27, and 30 proteins are ranked among the same top sets. Finally, to evaluate the statistical significance of the ranking results, we calculate the *p*-values by assuming that the underlying distribution of our problem follows a hypergeometric distribution [45] as follows:

8 $$ Px(k)=\frac{\left(\overset{K}{k}\right)\left(\overset{N\hbox{-} K}{n\hbox{-} k}\right)}{\overset{N}{n}}, $$

**Table 6:** Ranking result comparison for experimentally verified human saliva-secretory proteins

Top number	SVM	DeepLoc	CapsNet-SSP
1000	3 (0.168)	5 (0.025)	16 (5.28E-12)
2000	7 (0.042)	19 (2.13E-10)	24 (5.35E-16)
3000	7 (0.132)	27 (2.83E-15)	29 (7.28E-18)
4000	9 (0.121)	30 (1.65E-15)	33 (1.56E-19)

where *N*, which is the total number of human proteins, is 20,186, and *n* is the number of the selected top proteins. *K*, which is the number of experimentally verified human saliva-secretory proteins that do not overlap with the training set, is 37, and *k* is the number of proteins that are in the 37 saliva-secretory proteins and the top *n* predicted candidate proteins. The *p*-values of hypergeometric probability are 5.28E-12, 5.35E-16, 7.28E-18 and 1.56E-19 for such rankings using CapsNet-SSP, respectively. The ranking results show that CapsNet-SSP meets the requirement of statistical significance for predicting saliva-secretory proteins and has a better effect than the model based on SVM and the model based on the deep learning architecture DeepLoc.

Next, the function enrichment analysis is implemented by treating the entire set of human proteins as the background among the top 4000 proteins ranked by *S*-value, using the Database for Annotation, Visualization and Integrated Discovery (DAVID) [46] against the Gene Ontology and Kyoto Encyclopedia of Genes and Genomes (KEGG), which are pathway databases for understanding the cellular functions and subcellular locations of the predicted saliva-secretory proteins. The enrichment results show that the most significantly enriched biological processes, cellular components and molecular functions are ‘homophilic cell adhesion via plasma membrane adhesion molecules’, ‘extracellular region’ and ‘serine-type endopeptidase activity’. The most significantly enriched pathways are ‘extracellular matrix (ECM)-receptor interaction’, ‘complement and coagulation cascades’ and ‘protein digestion and absorption’ (see Additional file 2).

### Ranking known cancer biomarkers in saliva

To further confirm the effect of the proposed model on disease marker detection, we also rank the existing cancer biomarkers in saliva. We collect existing biomarkers in saliva from head and neck squamous-cell carcinoma (HNSCC) [24], oral squamous cell carcinomas (OSCC) [22], lung cancer (LC) [47] and breast cancer (BC) [7]. Table 7 shows the ranking results of the 25, 36, 11 and 10 biomarkers of secretory protein in HNSCC, OSCC, LC and BC, respectively. The results show that 19 (76.00%) of the 25 biomarkers in HNSCC, 34 (94.44%) of the 36 biomarkers in OSCC, 10 (90.91%) of the 11 biomarkers in LC, 9 (90.00%) of the 10 biomarkers in BR are ranked among the top 4000, respectively. Then, to evaluate the statistical significance of the ranking results, we calculate the *p*-values by assuming that the underlying distribution of our problem is a hypergeometric distribution. The *p*-values of hypergeometric probability are 2.01E-9, 4.55E-22, 8.16E-9 and 3.75E-6 in HNSCC, OSCC, LC and BC, respectively, among the top 4000. These ranking results show that saliva-secretory protein identification is statistically significant for existing cancer biomarkers in saliva.

**Table 7:** Comparison of the ranking results for different cancer biomarkers in saliva

Top number	HNSCC	OSCC	LC	BC
1000	7 (1.39E-4)	13 (7.31E-9)	6 (5.23E-6)	3 (0.010)
2000	11 (8.41E-6)	28 (6.73E-22)	7 (2.02E-9)	4 (0.011)
3000	15 (2.16E-7)	34 (2.03E-26)	9 (1.40E-6)	6 (0.001)
4000	19 (2.01E-9)	34 (4.55E-22)	10 (8.16E-9)	9 (3.75E-6)

## Discussion

Disease biomarkers play an important role in disease detection and the investigation of the mechanisms of disease occurrence and development. In particular, protein biomarkers in biological fluids have the potential to be measured and evaluated as indicators for differentiating normal and disease samples. In recent years, as the level of proteomic analysis has increased, a variety of clinical disease biomarkers have been discovered in body fluids. However, most of these biomarkers are found in blood, and few are detected in saliva. Saliva, as a source for detecting disease biomarkers, has obvious advantages over blood in terms of sample collection and clinical diagnosis.

Many studies have identified biomarkers for various diseases in saliva by comparative proteomic analysis of saliva samples from patients with specific diseases and controls. Comparing and quantifying proteomic data from saliva samples is a challenging task because saliva contains a large number of sparsely occurring proteins with large dynamic ranges that span several orders of magnitude. Consequently, due to the limitations of proteomic experimental techniques, the discovery of salivary biomarkers faces many problems. Thus, developing a method for accurately predicting human saliva-secretory proteins can solve these problems to some extent. However, few studies have focused on establishing computational models for predicting human saliva-secretory proteins, and all of these studies are based on conventional machine learning techniques, in which features are selected from feature sets and classifiers are constructed from training sets.

## Conclusions

In this study, we proposed an end-to-end model based on a deep learning framework that can accurately identify human saliva-secretory proteins solely from the sequence information of amino acids. The model consists mainly of a multilane Capsule Network with differently sized convolution kernels. The first step in constructing the model is to convert the input protein sequences into evolutionary profile matrices using PSI-BLAST. In addition, to address unbalanced dataset issues during training, the bagging ensemble learning method is applied to the training set. Then, the profile matrices of the training set are input into the proposed model to train the model parameters. Finally, the trained model is used to predict saliva-secretory proteins from the test set, and its performance is verified to be better than those of existing methods. Meanwhile, our model can satisfactorily detect the salivary protein biomarkers of cancer. The main contributions of this paper are the proposal of a deep-learning-based end-to-end prediction model that can accurately identify saliva-secretory proteins using only protein sequence information and achieving results that are statistically significant for existing cancer biomarkers in saliva.

## Methods

### Data collection

Several proteins that can be detected in saliva have been either curated in existing databases or reported in the literature. Therefore, we first collect proteins detectable in saliva from the Sys-BodyFluid database [48], which contains human body fluid proteins from previous proteome studies. The database includes 2161 proteins in saliva detected experimentally in eight salivary proteome studies. We also gather proteins in saliva from other salivary proteome studies [39, 49], resulting in 331 and 1166 additional proteins in saliva. Then, we filter these proteins using the data of experimentally validated secretory proteins from the secreted protein database (SPD) [43], the mammalian protein subcellular localization database (LOCATE) [44], and the Universal Protein Resource (UniProt) [36]. In addition, to avoid learning bias due to protein redundancies, we remove the proteins that have a mutual sequence similarity above 30% using the CD-HIT tool [50]. Finally, 450 proteins remain as the positive data (saliva-secretory proteins), of which 350 proteins are used as a positive training set while 100 proteins are used as the positive test set.

Because no proteins have been clearly reported as non-saliva-secretory proteins, generating the negative data is challenging. In this study, we use a method similar to that proposed by Cui et al [51], which chooses proteins from the Pfam families that do not contain proteins in the positive data. To reduce the influences of protein families that contain only a small number of proteins, we choose the proteins from families with at least ten proteins. For each family, three members are selected to construct the negative data. We also remove proteins from the negative data that have a mutual sequence similarity above 30% using the CD-HIT tool. As a result, 1850 proteins are selected as the negative data, of which 1750 are used as the negative training set, and the remaining 100 proteins are used as the negative test set.

### Input sequence encoding

A simple and widely used encoding method for protein sequences is one-of-K encoding (one-hot encoding), which consists of a matrix with *N* rows and *K* columns, where *K* is usually the number of amino acid types and *N* is the length of the input protein sequences [52]. In one matrix, each column corresponds to a type of amino acid, and each row indicates the location in a protein sequence, which is a *K*-dimensional vector with a value of 1 at the index corresponding to the amino acid and a value of 0 at all other positions. However, one-of-K encoding does not consider the evolutionary relationships among proteins. Therefore, in this study, the protein sequences are encoded to evolutionary profiles obtained by searching for the sequence in the ‘Uniref50’ database [53] using PSI-BLAST [54]. Specifically, the profile of each protein, which is generated using PROFILpro [55], is actually a normalized position-specific scoring matrix (PSSM) [56] based on the amino acid frequencies at every position of a multiple alignment.

In practice, the sequence lengths of different proteins are different. To reduce the training time, the maximum protein length is set to 1000, and for proteins with lengths less than 1000, the end of the matrix is padded with zeros. When a protein is longer than 1000, 500 amino acids from the beginning (N-terminus) and 500 amino acids from the end (C-terminus) of the protein are selected to avoid the loss of the sorting signals on the N-terminus and C-terminus. According to this rule, only 16.32% of human saliva-secretory proteins are truncated. Because most information on saliva-secretory proteins is stored at the beginning (N-terminus) and the end (C-terminus) of the sequence, this selection will retain the most information [24].

### Architecture design

**Fig. 6:**
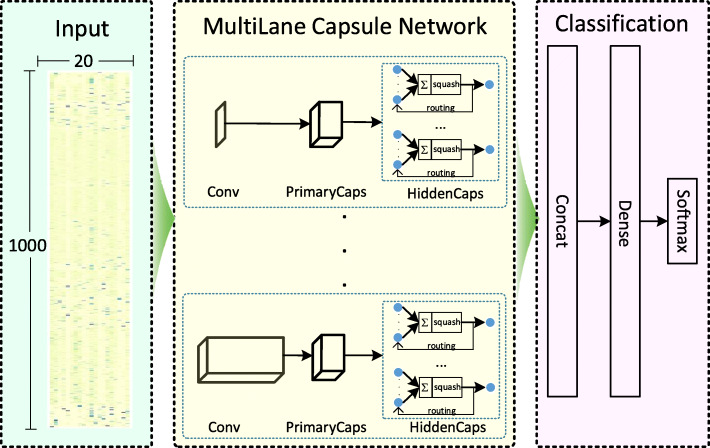
Architecture of the proposed model

The architecture of the proposed model based on a deep learning framework is shown in Fig. 6, and the architecture is summarized as follows. The input of the model is a 1000 × 20 normalized PSSM for each protein, which is used as input of the multilane Capsule Network (CapsNet). In this study, the multilane CapsNet contains eight lanes with differently sized 1D convolution kernels, and each CapsNet lane contains a convolutional layer (Conv), a convolutional capsule layer (PrimaryCaps) and a HiddenCaps layer.

The first convolutional layer (Conv) in each lane is designed to increase the representation power of the model. Each of them contains 10 1D convolution kernels of designated size with a stride of 1 and a ReLU activation function [57]. The kernel sizes of the eight lanes are set to 1, 3, 5, 9, 15, 21, 27 and 33, respectively. The first convolutional layer in each lane, which also uses the dropout technique with a dropout rate of 0.5 to prevent overfitting and to optimize model generalizability [58].

The PrimaryCaps layer is a convolutional capsule layer proposed by Sabour et al [59]. In this study, the PrimaryCaps layer in each lane is a 1D convolutional capsule layer with 8 channels of convolutional capsules. Each capsule consists of 16 convolutional units, each of which is the result of a size-1 1D convolution kernel with a stride of 9. In total, the PrimaryCaps layer in each lane contains [1000 × 8] capsule outputs (each output is a 16D vector), and each capsule in the [1000 × 1] grid shares its weight with others. Because the capsule length represents the probability of entity presentation, the convolutional units in the capsule layers require different activation functions than those used in convolutional neural networks [60]. The squashing activation function used in the PrimaryCaps layer scales the capsule lengths to [0, 1] as follows:

9 $$ {v}_j=\frac{\left\Vert {s}_j\right\Vert }{1\kern1em +\kern1em {\left\Vert {s}_j\right\Vert}^2}\frac{s_j}{\left\Vert {s}_j\right\Vert }, $$

where *s*_*j*_ is the input vector of capsule *j* and *v*_*j*_ is its output vector.

**Fig. 7:**
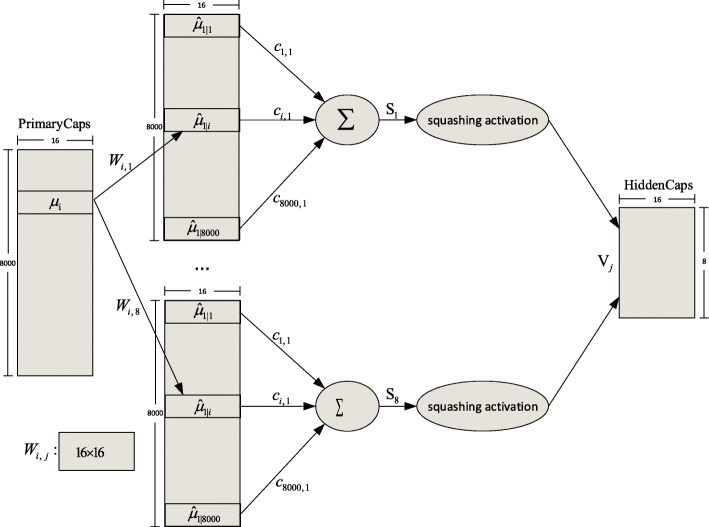
Computation between the PrimaryCaps and HiddenCaps layers of each group

The HiddenCaps layer in each lane consists of eight 16D capsules that map the input proteins to different states. The computation between the PrimaryCaps and HiddenCaps layers is shown in Fig. 7, where *μ*_*i*_, *i* ∈ [1, 8000] is a 16D vector in the PrimaryCaps layer, *W*_*i,j*_ is the weight matrix of an affine transformation, and *V*_*j*_ is a 16D vector obtained by the weighted sum *s*_*j*_ on all the outputs *μ*_*j* ∣ *i*_ of the PrimaryCaps layer. There are eight capsules (*V*_*j*_, *j* ∈ [1, 16]) in the HiddenCaps layer of each group that receive inputs from the outputs of the corresponding PrimaryCaps layer. The squashing activation function shown in Eq. 9 is applied to the HiddenCaps layer. *c*_*i,j*_ is the coupling coefficient determined by the iterative dynamic routing process, and these coefficients sum to 1 for the eight capsules of HiddenCaps in each group. To train the model by dynamic routing, five routing iterations are applied in this study. The complete dynamic routing algorithm can be found in the original CapsNet paper [59]. The outputs of the HiddenCaps layer in each group are merged in the concatenated layer. Then, the prediction outputs are produced by a fully connected dense layer with 128 units and a softmax layer for binary classification.

### Model training

During model training, all the deep learning models are trained using identical training strategies. The parameters of these deep learning models are optimized using the Adam stochastic optimization method [61] with the following parameters: a learning rate of 0.001, a decay rate for the first-moment estimate of 0.9, and an exponential decay rate for the second-moment estimate of 0.999. The applied loss function is cross-entropy loss between the true and predicted distributions of saliva-secretory proteins and non-saliva-secretory proteins. Both the proposed and compared models are executed on a workstation equipped with an Ubuntu 18.04.2 LTS operating system, an Intel Core i7-7800X CPU, 128 GB of RAM, and an NVIDIA GeForce RTX 2080 Ti GPU. All the deep learning models are implemented using Keras 2.2.4 and TensorFlow 1.13.1.

The dropout, early stopping and regularization strategies are used to prevent overfitting of the complex deep-learning model. The dropout strategy adds multiple dropout layers in the prediction model. During training, samples are taken from different sparse networks of index numbers. During testing, an unthinned network is used with a smaller weight to easily approximate the average of all the thinning network predictions [62]. Another strategy used to reduce overfitting is to stop early during training. Specifically, when the validation data loss is not reduced within some preset number of epochs, the training procedure is halted [63]. The third strategy for preventing overfitting is to use regularization in the deep learning model [64]. The optimization function contains two items: a loss term, which is used to measure the degree to which the model fits the data, and a regularization term, which is used to measure the model complexity and prevent overfitting. In this study, L2 regularization is adopted to prevent overfitting of the deep learning model.

### Bagging ensemble learning

The bagging ensemble learning method [33] is applied to the training set to reduce the impact of unbalanced data. By training models on several selected balanced subsets, we obtain several independent classifiers. Then, the final prediction is calculated by averaging the results of these independent classifiers. The bagging ensemble learning algorithm used to train our proposed model is given below:

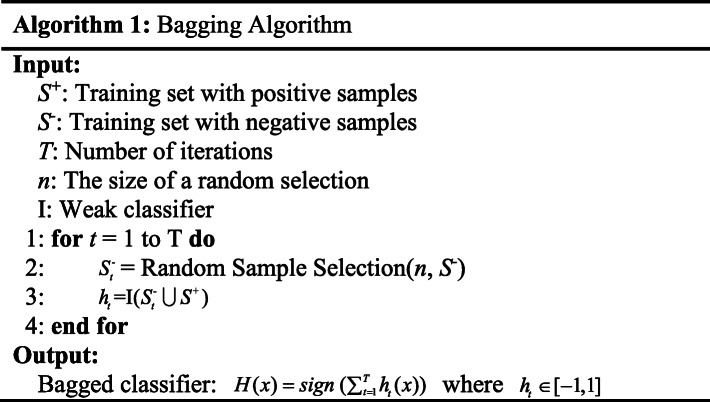



Here, *S*^*+*^ contains 350 saliva-secretory proteins, and *S*^*−*^ contains 1750 non-saliva-secretory proteins. The number of iterations *T* is 10, and the size of the random selection *n* is 350.

## Supplementary information

**Additional file 1.** Thirty-Seven human saliva-secretory proteins that do not overlap with training set. These proteins are collected using the LC-MS/MS analyses reported in the literature and databases of SPD, LOCATE and UniProt. Then, the proteins in the training set are removed.

**Additional file 2. **Function enrichment analysis of top 4000 proteins. The function enrichment analysis is implemented by treating the entire set of human proteins as the background among the top 4000 proteins ranked by *S*-value, using DAVID against the GO and KEGG pathway.

## Data Availability

The pipeline of the model, prediction data and results can be accessed at the following URL: http://www.csbg-jlu.info/CapsNet-SSP/.

## Abbreviations

CapsNet: Capsule network
HNSCC: Head and neck squamous-cell carcinoma
PSI-BLAST: Position-specific iterative basic local alignment search tool
SPD: Secreted protein database
LOCATE: Mammalian protein subcellular localization database
UniProt: Universal protein resource
PSSM: Position-specific scoring matrix
MCC: Matthews correlation coefficient
ROC: Receiver operating characteristic
AUC: The area under the receiver operating characteristic curve
TPR: True positive rate
FPR: False positive rate
KNN: K-nearest neighbor
AdaBoost: Adaptive boosting
CNN: Convolutional neural network
BLSTM: Bidirectional long short-term memory network
DAVID: The database for annotation, visualization and integrated discovery
KEGG: Kyoto encyclopedia of genes and genomes
OSCC: Oral squamous cell carcinomas
LC: Lung cancer
BC: Breast cancer
